# Utility of tryptase genotyping in the screening, diagnosis, and management of systemic mastocytosis

**DOI:** 10.3389/falgy.2025.1599358

**Published:** 2025-05-27

**Authors:** Jeremy C. McMurray, Brandon J. Schornack, Joaquin Villar, Tracy I. George, Nathan A. Boggs

**Affiliations:** ^1^ Allergy & Immunology Service, Walter Reed National Military Medical Center, Bethesda, MD, United States; ^2^ Department of Pediatrics, Uniformed Services University, Bethesda, MD, United States; ^3^ Henry M. Jackson Foundation for the Advancement of Military Medicine, Bethesda, MD, United States; ^4^ Center for Military Precision Health, Uniformed Services University, Bethesda, MD, United States; ^5^ ARUP Laboratories and Huntsman Cancer Institute, Department of Pathology, University of Utah, Salt Lake, UT, United States; ^6^ Department of Medicine, Uniformed Services University, Bethesda, MD, United States

**Keywords:** systemic mastocytosis (SM), tryptase genotyping, basal serum tryptase (BST), hereditary alpha tryptasemia, tyrosine kinase inhibitors (TKIs)

## Abstract

Tryptase genotyping has an expanding role in the screening, diagnosis, and management of patients with systemic mastocytosis (SM). Reference ranges for basal serum tryptase (BST) based on increased *TPSAB1* gene copy number can guide whether a patient's BST value is normal according to their specific tryptase genotype. Patients with an elevated BST based upon their tryptase genotype should be offered a bone marrow biopsy with sample evaluation by a hematopathologist. Tryptase genotyping is required when assessing patients for the WHO minor criterion, BST > 20 ng/ml, especially in those with monoclonal mast cell activation syndrome, bone marrow mastocytosis (BMM), and indolent systemic mastocytosis (ISM) when the major criterion is not met. Additionally, in patients with non-advanced SM, tryptase genotyping helps determine whether a patient with hereditary-alpha tryptasemia (HαT) has BMM with a BST < 125 ng/ml or fulfills the B-finding of BST > 200 ng/ml through application of a correction factor. Understanding a patient's BST level based upon their tryptase genotype also is helpful in guiding when to pursue a repeat bone marrow biopsy in patients with SM treated with a tyrosine kinase inhibitor (TKI). However, TKIs have variable KIT D816V as well as wild type KIT inhibition. Given this variable KIT inhibition, ongoing and future clinical trials with selective TKIs should report whether patients with SM and HαT experience normalization or persistent elevation of BST values as this is essential in understanding the expected treatment response and when to assess for pathological remission in the bone marrow.

## Introduction

Mast cells (MCs) are a group of tissue-resident white blood cells characterized by metachromatic granules that contain mediators released during the early- and late-phase allergic responses. Tryptases are serine proteases that were first identified in MC granules in 1981 and they are the most abundant protein mediator in MC secretory granules (1–3). In humans, MCs can be identified through granular cytoplasmic expression of tryptase by immunohistochemistry and MC subpopulations in various tissues can be further delineated based upon the granular cytoplasmic co-expression of both tryptase and a second serine protease known as chymase (4). Tryptase and other MC mediators, including chymase, histamine, heparin, prostaglandins, leukotrienes, cytokines, and acid hydrolases, are released from secretory granules during degranulation and collectively contribute to allergic disease. MC mediators may affect the expression and stability of one another. For instance, exogenous histamine induces a concentration-dependent increase in tryptase (5). Conversely, exogenous tryptase can also induce a concentration-dependent increase in histamine (6). Heparin combined with an acidic pH serves to stabilize tetrameric tryptase via four histidine residues found in tryptase (7–10). An individual role for tryptase in human disease has not yet been established (11). By contrast, histamine has been demonstrated to cause cardiovascular instability when infused directly into human subjects and histamine receptor antagonists are widely used in the treatment of allergic disease (12). There is no clear role for tryptase or other MC mediators in human health (13).

Elevations in basal serum tryptase (BST) values occur at increased frequency in patients with the myeloid neoplasm systemic mastocytosis (SM) (11, 12). SM is characterized by the abnormal accumulation of neoplastic MCs in one or more organ systems. BST values ≥20 ng/ml are incorporated into the 2022 World Health Organization (WHO) and International Consensus Classification (ICC) diagnostic criteria for SM (13, 14). Additionally, BST values are used as a disease burden biomarker corresponding to the quantity of neoplastic mast cells in patients with SM. BST >200 ng/ml is a marker of high neoplastic MC burden. Serial monitoring of BST values is an important aspect of SM disease management in patients treated with tyrosine kinase inhibitors (15). It has recently been determined that the most common etiology for elevated BST in the general population is due to copy number variation at the tryptase locus gene, *TPSAB1,* rather than SM and this has led to refinements in SM screening (11, 16). Further, BST reference ranges specific to an individual's tryptase genotype, based on copy number variation at *TPSAB1*, have been reported (17). Thus, tryptase genotyping is becoming an important clinical tool in the management of patients with SM. Here, we explore the specific use of tryptase genotyping for SM screening, application of SM diagnostic criteria and subtype determination, and guiding SM management in patients treated with tyrosine kinase inhibitors.

### Tryptase locus

**Figure 1:**
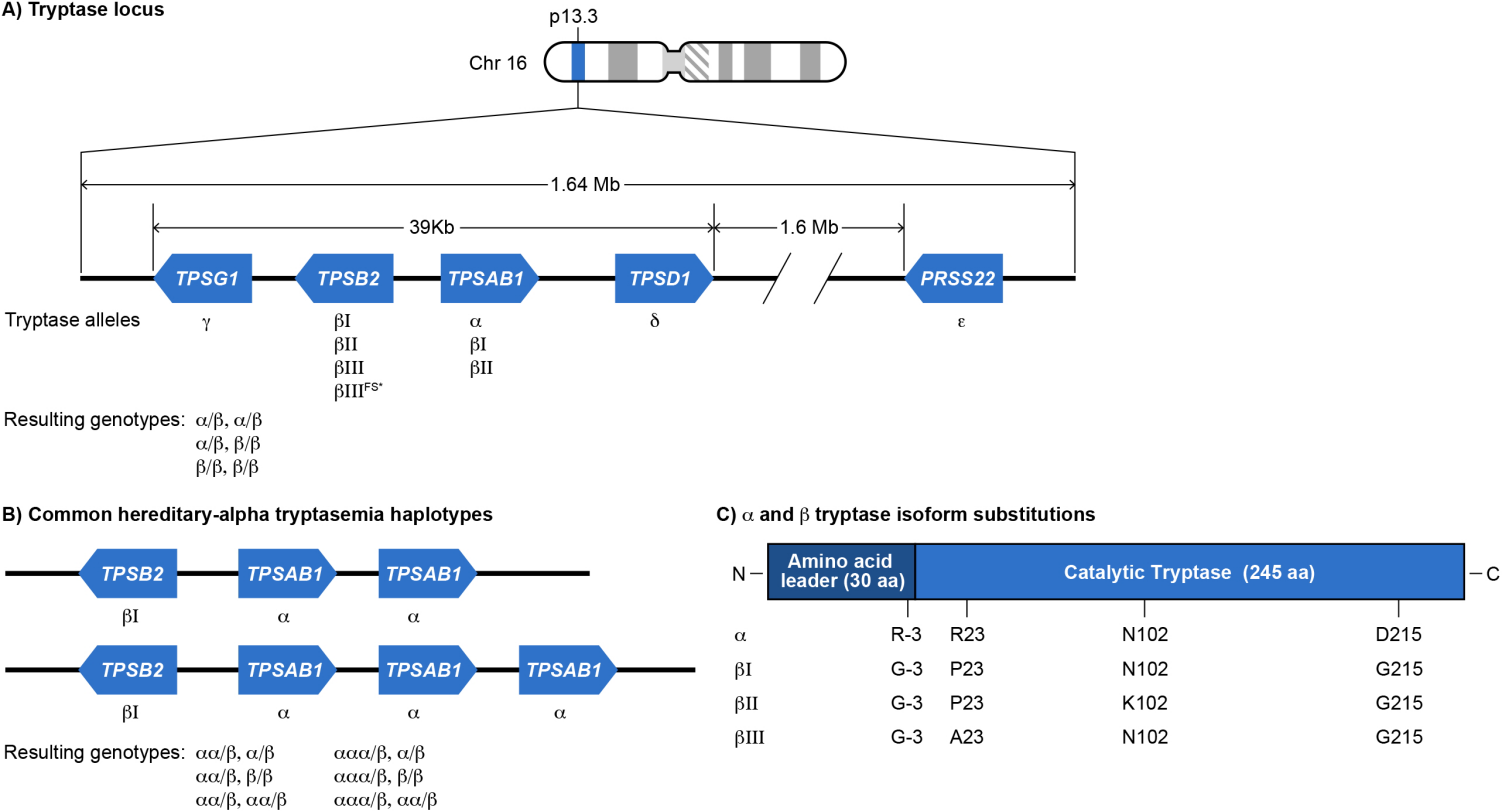
**(A)** Schematic of tryptase gene cluster and its wild type genotypes. Tryptase locus on chromosome 16p13.3 comprises a homologous cluster of genes: *TPSAB1*, *TPSB2*, *TPSD1* and *TPSG1*. *TPSAB1* and *TPSD1* are located on the positive strand of DNA, while *TPSB2* and *TPSG1* are on the negative strand. (23) An additional human tryptase (ε) encoded by PRSS22 also exists on 16p just outside of this cluster on the negative strand. *TPSAB1* and *TPSB2* encode soluble tryptases (α and β). The resulting normal *TPSAB1*/*TPSB2* genotypes are shown (specific β alleles are not indicated). **(B)** Hereditary-α tryptasemia (HαT) alleles and genotypes. The HαT genetic trait is defined as one or more tandem increased copies of the *TPSAB1* α allele. (97) Tandem *TPSAB1* α allele duplications and triplications are the most commonly reported copy number variants that underly HαT, though even higher tandem copy numbers can occur. (16) The resulting most common HαT *TPSAB1*/*TPSB2* genotypes are shown (specific β alleles are not indicated). **(C)** Each α− and β-tryptase allele encodes a 275-amino acid peptide with a 30-amino acid-leader sequence and a mature catalytic portion of 245 amino acids. Each of these isoforms is highly similar, being at least 97% identical. (98) The key amino acids differentiating each isoform are Arg/Gly at −3, Pro/Arg 23, Asn/Lys at 102 and Asp/Gly at 215. (26, 27). *bIIIFS-tryptase p.M123Dfs*14 is inactive.

The tryptase locus found at chromosome 16p13.3 is a 1.64 megabase region containing tryptase genes encoding soluble and membrane-bound serine proteases (Figure 1A). Tryptase genes are part of an ancient gene family that arose in non-mammalian vertebrates (18). From telomere to centromere, these genes include *TPSG1*, *TPSB2*, and *TPSAB1* (19, 20). *TPSB2* and *TPSAB1* are >90% similar in sequence while *TPSG1* is only 47% similar to *TPSB2* (20, 21). Additional related genes of reduced function or pseudogenes are distal to *TPSAB1* towards the centromere including *TPSD1* and *PRSS22* (22, 23). The tryptase locus genes were first cloned and described in humans in 1999 (20, 21, 24). The *TPSG1* gene encodes γ tryptase isoforms. γ tryptases have a hydrophobic tail that results in them being membrane-bound (21, 24). Genomes of non-mammalian vertebrates encode homologs of *TPSG1,* but not *TPSB2* or *TPSAB1,* suggesting that membrane-bound tryptase isoforms evolved first (18). The *TPSB2* and *TPSAB1* genes likely arose in mammals. They encode soluble tryptases that localize to MC granules as tetramers and are also constitutively secreted as pro-tryptase monomers (25). The *TPSB2* gene encodes β tryptase isoforms. The three *TPSB2* alleles described in humans includes the βI minor allele, βII major allele, and βIII major allele. Approximately 20% of the population may have a frameshifted *TPSB2* βIII null allele (βIII^FS^) (26). The *TPSAB1* gene encodes either the α major allele, βI major allele, or βII minor allele. β tryptase homotetramers are functional serine proteases while homotetrameric α tryptases are likely non-functional based on crystal structure and biochemical analysis, due to amino acid substitutions at residues −3 and 215. The Arg-3Gln substitution in the N-terminal pro-peptide of α tryptase evolved recently and leads to faulty zymogen activation. The Gly215Asp substitution in the catalytic primary specificity pocket leads to reduced substrate binding and flawed catalytic activity (27–29). αβ heterotetramers occur in proportion to the number of α alleles present. The protease activity of heterotetramers has been incompletely characterized, although one study showed that heterotetramers *in vitro* can activate protease activated receptor-2 and cleave EGF-like module—containing mucin-like hormone receptor-like 2 (30). The mutation causing the Gly215Asp substitution in α tryptase arose in Old World monkeys after they split from New World monkeys. This mutation also arose before the split of α and β alleles at *TPSAB1* (18). Site directed mutagenesis to swap Gly for Asp at residue 215 was sufficient for α tryptase to gain βII enzymatic activity (29).

### Hereditary-alpha tryptasemia (HαT) genetic trait

Tandem increased copy number of the α allele of *TPSAB1* on one or more chromosomes is known as the HαT genetic trait and was first identified in 2016 (Figure 1B) (16). The tandem α alleles in HαT have an expanded promoter repetitive element that is linked to increased α tryptase expression (17). It is not clear how many founder events for the HαT genetic trait may have occurred during human evolution, in which human populations these founder events occurred, and whether the trait occurred due to genetic drift or positive selection. Two studies have reported on the prevalence of HαT in a general population sample. In 2016, when HαT was first reported, the authors assessed a sample of 98 individuals and found 8 (8.2%) that had both elevated BST values and HαT. A subsequent study in the UK of 423 individuals found that 22 (5%) had HαT (16, 31). BST references ranges based upon the number of tandem *TPSAB1* α alleles were reported in 2021 (Table 1) (32). Modeled BST reference range values were subsequently reported in 2023 and showed that each additional tandem *TPSAB1* α allele contributes around 10 ng/ml to BST on average (17). The genes at the tryptase locus, particularly *TPSB2* and *TPSAB1*, are under strong linkage disequilibrium and are inherited as haplotypes (26). There are two major tryptase locus haplotypes of *TPSB2*-*TPSAB1* for individuals who do not have HαT that occur at a frequency of >15% and they are βII-α and βIII-βI. There are additional minor haplotypes that occur at a frequency of <15% (26). The major haplotype for *TPSB2*-*TPSAB1* for individuals who have HαT appears to be βI-α^DUP^ (17).

**Table 1:** Predicted basal serum tryptase median, range, and upper 99% interval according to TPSAB1 α allele copy number.

# Additional *TPSAB1* copy number	Tryptase genotypes	Predicted median BST (range) (ng/ml)	Predicted upper 99.5% BST value (ng/ml)
0	ββββ, αβββ, ααββ, αββββ, βββββ, βββ, αββ	4.1 (0–10.4)	11.4
1	ααβββ, αααββ	13.6 (6.5–33.9)	36.2
2	ααααββ, αααβββ	22.5 (10.5–39.5)	62.2
3	αααααββ	27.3 (23.4–40)	88.8
4	αααααβββ, ααααααββ	37 (25.5–62.7)	115.9
6	ααααααααββ	87 (NA)	171.2
10	ααααααααααααββ	133 (110–156)	285.1

Adapted from Chovanec et al. (17). BST, basal serum tryptase.

### Assays for both tryptase genotyping and serum tryptase

Comparison of an individual's tryptase genotype to their BST value is necessary to determine whether their BST is elevated. Tryptase genotyping refers to clinical assays designed to report a summation of the α and β tryptase allele copy numbers encoded at the *TPSAB1* and *TPSB2* genes. A digital droplet polymerase chain reaction (ddPCR) assay using primer and probe sets for α- and β-tryptase to quantify α- and β-tryptase allele copy number was developed in 2016 (16). This method is now available in a few select commercial laboratories in the United States. The α and β allele copy number reported in ddPCR assays typically allows one to determine tryptase haplotypes for both chromosomes in the individual tested. However, in some cases, α and β copy number may correspond to multiple possible haplotypes instead of only one haplotype. Additionally, the β tryptase allele copy number reported does not distinguish between βI, βII, βIII, or βIII^FS^ alleles.

Since 2016, three additional clinical tryptase genotyping assays have been reported. Two studies have reported a multiplex ddPCR assay, which allows for quantification of α- and β-tryptase copy counts in a single reaction. Compared to the original ddPCR assay, the multiplex ddPCR may have a lower cost and runtime, and a strong correlation with BST and overall accuracy (33, 34). A second reported method is the target amplicon next-generation sequencing (NGS) assay, which utilizes machine learning models to identify polymorphisms at *TPSAB1* and *TPSB2* genes (35). In this study, this NGS assay accurately estimated 96% of both α and βIII^FS^ tryptase alleles, and 94% of extra α alleles on *TPSAB1* (35). Neither the multiplex nor NGS methods are commercially available.

Several serum tryptase assays have been developed over time and a few are described here. The first assays utilized a low sensitivity mouse monoclonal G5 anti-tryptase antibody which can detect linear epitopes on denatured tryptase. Initially, a sandwich enzyme-linked immunosorbent assay (ELISA) utilizing the G5 capture antibody with a goat polyclonal anti-tryptase detector antibody was developed (36). Then, a sandwich radioimmunoassay was developed utilizing the G5 capture antibody with a mouse monoclonal G4 detector antibody (37). The assays developed next utilize the more sensitive mouse monoclonal B12 antibody. The B12 antibody detects epitopes of tetrameric and denatured linear tryptase. Initially, an ELISA was developed that utilized the B12 capture antibody with a biotin-G4 detector antibody (38). Clinical serum tryptase testing now utilizes quantitative fluorescent-based immunoassays (e.g., ImmunoCAP) using the B12 capture antibody to measure total tryptase, reported as one value corresponding to the sum of both mature tetrameric tryptase and monomeric α- and β-protryptases.

### Systemic mastocytosis

There are three types of mastocytosis: cutaneous mastocytosis (CM), systemic mastocytosis (SM), and MC sarcoma. The three subtypes of CM include maculopapular CM (MPCM), diffuse CM, and mastocytoma (39). MPCM can be polymorphic or monomorphic. SM subtypes include bone marrow mastocytosis (BMM), indolent SM (ISM), smoldering SM (SSM), aggressive SM (ASM), SM with an associated hematologic neoplasm (SM-AHN), and MC leukemia (MCL). BMM, ISM, and SSM are non-advanced (non-AdvSM) and ASM, SM-AHN, and MCL are advanced subtypes (AdvSM). Well-differentiated SM is a morphologic pattern occurring in any SM subtype and is characterized by enlarged round and well-granulated MCs (13). A diagnosis of SM is established when at least 1 major and 1 minor or 3 minor criteria are met, as detailed in the 5th edition of the WHO diagnostic criteria (Table 2) (13). Monoclonal mast cell activation syndrome (MMAS) occurs when the WHO major criterion is not met and only two minor criteria are met. SM subtypes are determined based on the presence of B (disease “burden”) and C (need for “cytoreductive” treatment) findings (Table 3) (13). Patients with BMM have no B or C findings, the BST should be <125 ng/ml, and they have no skin involvement. ISM patients can have only 1 B finding, SSM patients must have 2 or more B findings, and ASM patients have one or more C findings. MCL requires ≥20% neoplastic mast cells in the BM aspirate (13). Greater than 90% of patients with SM have the activating mutation *KIT* c.2447 C > T p.D816V (40).

**Table 2:** 2022 World Health Organization SM criteria.

Major SM Criterion
1. Multifocal dense infiltrates of MCs (≥15 MCs in aggregates) in BM biopsies and/or in sections of other extracutaneous organ(s)
Minor SM Criteria
1. ≥25% of all MCs are atypical cells (type I or type II) on BM smears or are spindle-shaped in MC infiltrates detected on sections of BM or other extracutaneous organs
2. *KIT* point mutation at codon 816 or in other critical regions of *KIT* in the BM or another extracutaneous organ
3. MCs in BM or blood or another extracutaneous organ exhibit CD2 and/or CD25 and/or CD30
4. Baseline serum tryptase level >20 ng/ml (in case of an unrelated myeloid neoplasm, an elevated tryptase is not valid as an SM criterion. In case of known HαT, tryptase level should be adjusted)
If at least 1 major and 1 minor or 3 minor SM criteria are fulfilled, the diagnosis of SM can be established

Adapted from Khoury et al. (13).

**Table 3:** 2022 World Health Organization B- and C-findings.

B Findings:
1. High MC burden showing infiltration in BM ≥ 30% and/or serum tryptase ≥200 ng/ml and/or *KIT* p.D816V VAF ≥ 10% in BM or peripheral blood
2. Signs of myeloproliferation and/or myelodysplasia not fulfilling criteria for AHN
3. Hepatomegaly on palpation or imaging (ultrasound, CT, or MRI) without ascites or other signs of organ damage and/or splenomegaly on palpation or imaging without hypersplenism and/or lymphadenopathy on palpation or imaging (> 20 mm)
C Findings:
1. Cytopenia(s) (one or more found): absolute neutrophil count <1 × 10^9^/L, hemoglobin <10 g/dl, platelet count 100 × 10^9^/L
2. Hepatopathy: ascites and elevated liver enzymes ± hepatomegaly or cirrhotic liver ± portal hypertension
3. Palpable splenomegaly with hypersplenism ± weight loss ± hypoalbuminemia
4. Malabsorption with hypoalbuminemia ± weight loss
5. Large-sized osteolysis (≥ 20 mm) ± pathologic fracture ± bone pain

Adapted from Khoury et al. (13). AHN, associated hematological neoplasm; MC, mast cell; BM, bone marrow; VAF, variant allele frequency.

SM is rare with a prevalence of 0.9–1.7 per 10,000 individuals, of which ISM is the most common subtype (41–44). ISM with skin lesions and BMM together represent approximately 82%–91% of all SM patients (42, 45, 46). Other less prevalent subtypes include SSM occurring in 4.3%–7.1%, SM-AHN in 3.1–13.5%, ASM in 5.1%–9.7%, and MCL in 1-7%–4.8% of all SM patients (41, 42, 45–49). Each SM subtype has different prognostic implications. Patients with SSM have a higher risk of progression to AdvSM at 9.4%–15% compared to ISM and BMM at 4.9% and 1.7%, respectively, over a median follow-up period of 2.0–4.3 years (48, 50, 51). A large cohort of patients with a median follow-up time of 62 months demonstrated a 10-year progression-free survival rate of 100% in BMM, 98.1% in ISM with skin lesions, 87.4% in SM-AHN, 62.5% in SSM, and 55.6% in ASM (41). Although patients with ISM generally have a near-normal life expectancy, the median survival in advanced forms is significantly less with 3–5 years for SM-AHN, 3–4 years for ASM, and 0.5–1.6 years for MCL (50, 52–54).

Heterogeneity of SM clinical presentations and access to high complexity testing likely contributes to diagnostic delays with a median time to diagnosis of 7 years across all subtypes, with delays being highest in patients with non-AdvSM (55). Patients may experience frequent and debilitating cutaneous, gastrointestinal, musculoskeletal, and neurocognitive symptoms that impact quality of life (56–59). Around half of SM patients report a history of one or more anaphylaxis episodes and the grade of anaphylaxis is usually severe. Anaphylaxis in SM patients can be triggered by Hymenoptera envenomation, foods, drugs, or be idiopathic (53, 60–62). Osteoporosis and fragility fractures may occur in up to 30% and 50% of SM patients, respectively. Osteoporosis and fractures are more prevalent in ISM than AdvSM, with the latter showing more osteosclerosis (46, 63–66). Skin lesions are also reported in around half of SM patients (46, 51, 67). Additionally, nearly half of patients with non-AdvSM may present with BST values <20 ng/ml. SM patients with low BST values may experience delays in obtaining diagnostic bone marrow (BM) biopsies due to a perception that their BST values are too low to be consistent with SM. This is most often true of patients with ISM and BMM, and less commonly with SM-AHN (67–70). Access to high-complexity testing, including mast cell flow cytometry, high-sensitivity quantitative *KIT* p.D816V assays, and tryptase genotyping is not uniform across institutions. Overcoming these barriers is important as there are now disease-modifying selective TKIs that improve the morbidity and prognosis of SM.

### Tryptase genotyping in SM screening

**Figure 2:**
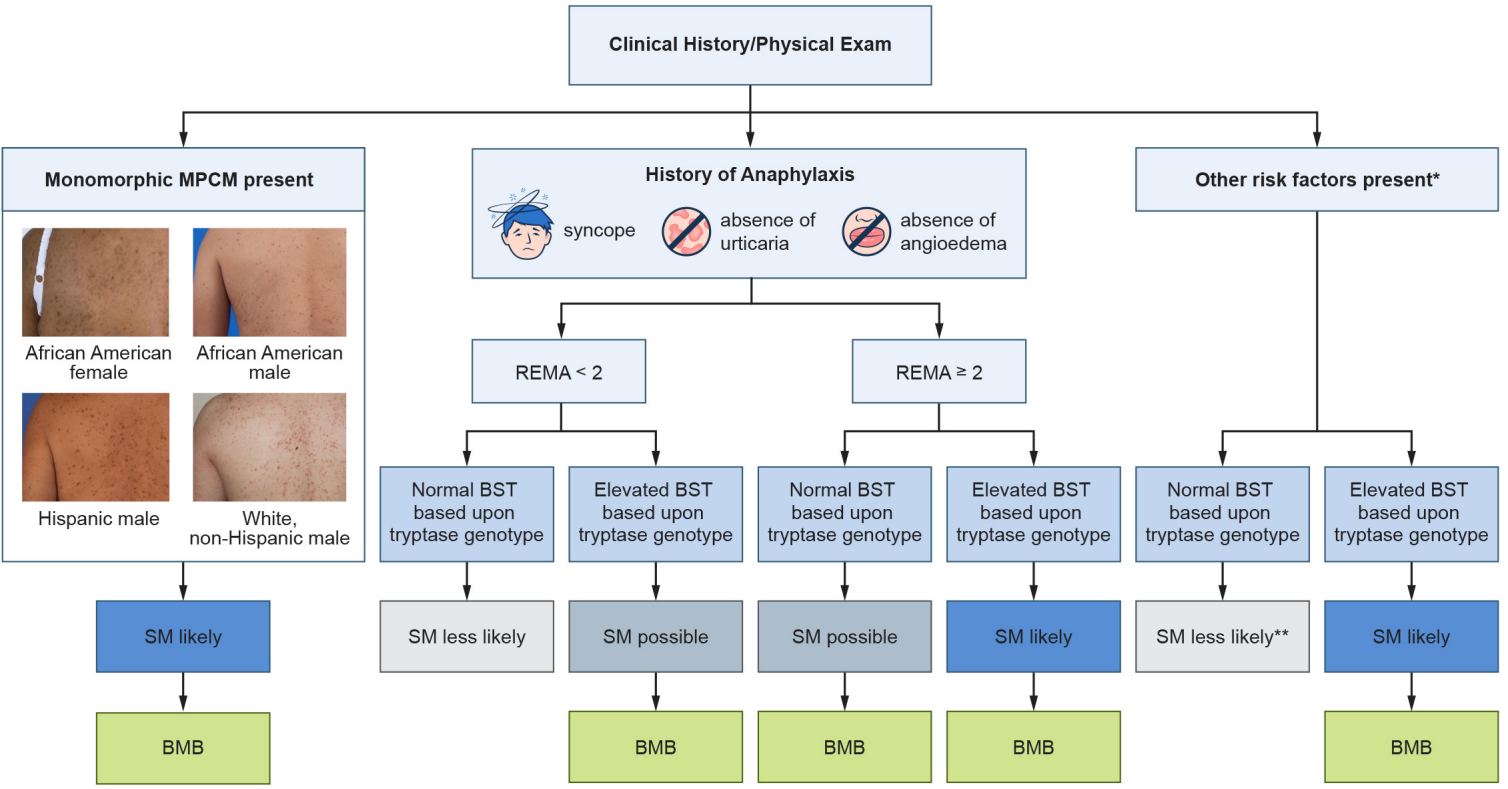
Screening algorithm for systemic mastocytosis. Clinicians should assess for a history of anaphylaxis, perform a skin exam, and assess for other SM risk factors. Tryptase genotyping is especially important in patients with anaphylaxis as well as patients with other SM risk factors. Additionally, some patients with monomorphic MPCM may be lacking a confirmatory skin biopsy and an elevated BST based upon an individual's tryptase genotype could obviate the need to perform a skin biopsy. *Other risk factors include flushing, unexplained osteoporosis, pathologic fracture, splenomegaly, and blood count abnormalities. Additional less specific risk factors include headache, diarrhea, fatigue, difficulty concentrating, and poor memory. Adapted with permission from “Screening approach to clonal mast cell disease in patients with Hymenoptera venom allergy (HVA)” by Nathan A. Boggs, Ilaria Tanasi, Karin Hartmann, Roberta Zanotti, and David Gonzalez-de-Olano, licensed under CC BY-NC-ND.

Screening for SM has historically been challenging due to the disease's variable clinical presentation. Advances in the last 15 years have led to an optimized screening strategy (Figure 2). The first milestone occurred in 2010 with the development of a clinical and laboratory scoring system known as the Red Española de Mastocitosis (REMA) score (71). Eighty-three patients with a history of anaphylaxis, but no mastocytosis-in-skin, were assessed for variables that might predict the presence of SM. A multivariate analysis demonstrated that male sex, BST values >25 ng/ml, as well as clinical manifestations during anaphylaxis of syncope or presyncope, and the absence of urticaria and angioedema were linked to SM. Syncope or presyncope during an episode of anaphylaxis were the most tightly linked clinical findings. The specificity of the REMA score is 74% (67, 72). The REMA score sensitivity is challenging to determine since approximately half of patients with SM may have no history of anaphylaxis. Among SM patients who experience at least one episode of anaphylaxis but without skin involvement, the sensitivity of the REMA score is 87% (72). Among all SM patients, regardless of history of anaphylaxis or skin involvement, the sensitivity of the REMA score is 34% (67).

The second major change to the overall SM screening approach was proposed in 2014. An analysis of 59 patients with adult-onset MPCM demonstrated that 97% of these patients had SM, indicating that adult-onset MPCM is specific for systemic disease (73). It is not clear whether any of these adult patients might have had polymorphic MPCM since polymorphic MPCM is nearly always found in children and polymorphic and monomorphic terminology was not in use at the time of the study. Notably, around half of SM patients may have skin involvement and most of these patients are thought to have adult-onset MPCM. A subsequent study in 2024 demonstrated that monomorphic MPCM is tightly linked to SM with a specificity of 97% (67).

Tryptase genotyping became clinically available around 2020 in the United States. The first study to assess the benefit of tryptase genotyping in SM screening was published in 2022 and assessed a group of 58 patients. SM and other myeloid neoplasms were found to be enriched in patients with elevated BST values who did not have HαT. Specifically, in patients with a BST value ≥11.5 ng/ml, 63% had HαT, 20% had myeloid neoplasms, and 12% had CKD. These results suggested that myeloid neoplasms are much more likely to be present in those with elevated BST, when HαT is absent (11). A second study to assess the benefit of tryptase genotyping in patients with elevated BST who do not have CKD was published in 2023. A group of 409 patients with elevated BST were screened for HαT and myeloid neoplasms. Of these 409 with BST ≥11.5 ng/ml, 74% had HαT and 29% had SM or another myeloid neoplasm (17). Finally, a third study assessed screening testing accuracy among a variety of screening tests including an elevated BST based upon tryptase genotype, BST ≥11.5 ng/ml, BST ≥20 ng/ml, and REMA combined with an elevated BST based upon tryptase genotype. This study demonstrated that the single most accurate screening test for SM, based on Youden's index which is a value based on the sum of the sensitivity and specificity, was elevated BST based upon tryptase genotype with a sensitivity of 84% and specificity of 90%. Further, it was found that the REMA score combined with elevated BST based upon tryptase genotype had a substantially improved specificity over the REMA score alone (70). An elevated BST based upon an individual's tryptase genotype may also be helpful when there is uncertainty in the diagnosis of anaphylaxis due to absence of a trigger (e.g., Hymenoptera venom) or when anaphylaxis occurs with mild symptoms such as lightheadedness rather than syncope.

SM is linked to other clinical manifestations that, on their own, may be less specific for SM, including osteoporosis, large osteosclerotic bone lesions, flushing, chronic diarrhea, fatigue, frequent headaches, and more. In 2024, one study assessed the combination of unexplained osteoporosis in patients with either elevated BST or symptoms of MC activation (74). The authors noted that common causes of osteoporosis had been excluded though it was not explicitly stated which causes those were. Nonetheless, the authors assessed 139 patients and showed that SM is more common in patients with osteoporosis and BST ≥11.5 ng/ml, if they also had BST >19 ng/ml, vertebral fractures, and were <54 years old. They developed several scoring systems designed to predict who might have SM in those with unexplained osteoporosis and elevated BST. The scoring test has a sensitivity of 71% and specificity of 67%. When BST >19 ng/ml was removed and replaced with an elevated BST based upon genotype, the scoring system had an improved sensitivity of 87% and specificity of 76%.

Some centers may not yet have access to tryptase genotyping. Measurement of MC mediators or metabolites, including leukotriene E_4_ (LTE_4_), N-methylhistamine (NMH), and 11 β-prostaglandin F2 α (BPG), in urine samples represent another means to assess the pretest probability of SM (75–77). The most specific urinary mediator was found to be NMH with a specificity of 88% (78). It is not clear how sensitive NMH and other urinary mediators are in SM screening in patients with a low disease burden as prior studies excluded patients with low BST values or did not describe the SM disease burden of their cohort. The relative accuracy of urinary mediators in predicting SM has not yet been directly compared to BST combined with tryptase genotyping, monomorphic MPCM, or the REMA score.

### Importance of tryptase genotyping in SM diagnostic testing and subtyping

The diagnosis of SM relies on determining whether the WHO major and minor criteria are met by an experienced hematopathologist. Notably, the minor criterion of BST ≥20 ng/ml was first introduced into the WHO SM diagnostic criteria in 2001, based on the idea that most patients with SM have BST values >20 ng/ml (79). Data supporting a specific diagnostic BST cutoff of >20 ng/ml, rather than other elevated BST values, is limited. One study showed that as many as 50% of SM patients may have BST values <20 ng/ml (67). The 5th edition of the WHO classification published in 2022 recommended adjustment of BST in case of HαT, although a specific manner of adjustment was not provided (13). The 2022 International Consensus Classification (ICC) of myeloid neoplasms and acute leukemias did not include adjustment of BST in the case of HαT (14). Neither the WHO nor the ICC included adjustment of BST in the case of HαT for a BST >200 ng/ml, which is a B-finding, or for a BST >125 ng/ml in patients with non-AdvSM and no skin lesions. Predicted median and upper 99.5% BST values for incremental tandem *TPSAB1* α allele replications has been reported (17). An online calculator that adjusts BST using a correction factor based on *TPSAB1* α allele replication number also has been published (https://bst-calculater.niaid.nih.gov) (17). Finally, a recent proposal has recommended harmonization of diagnostic criteria across organizations and to adjust for HαT by dividing the BST by 1 plus the extra copies of the *TPSAB1* α allele in order to determine whether SM criteria are met and whether the B-finding of BST >200 ng/ml is present. The importance of tryptase genotyping to determine whether an SM subtype is BMM in a patient with non-AdvSM, no B or C findings, and without skin lesions when the BST is >125 ng/ml was not specifically addressed (80).

There are several factors to consider when an SM diagnosis or subtype depends specifically on BST values. First, greater than 95% of patients with SM have disease driven by the *KIT* p.D816V mutation. MC spindling and expression of CD25 are found in nearly all cases where *KIT* p.D816V is detected in either peripheral blood or BM, even when the VAF approaches the limit of detection using clinically validated high-sensitivity PCR clinical assays (81, 82). Thus, BST values are not typically required to establish an SM diagnosis unless suboptimal diagnostic testing is employed. Second, it would be advantageous for allergy, hematology, and pathology teams to consistently employ tryptase genotyping in every suspected case of SM. This may be challenging in the short term as some centers currently do not have access to tryptase genotyping. It is worth noting that SM patients with relatively high BST values and a higher rate of HαT occurrence may be preferentially referred for BM biopsies compared to SM patients with lower BST values and this supports the need to employ tryptase genotyping in all SM diagnostic evaluations (67). Third, it is likely best to avoid the use of BST ≥20 ng/ml as a minor criterion, BST > 200 ng/ml as a B-finding, and BST >125 ng/ml for BMM/ISM subtype determination in the absence of tryptase genotyping. The impact of not having tryptase genotyping available when BST is >200 ng/ml (B-finding of increased MC burden) is partially mitigated by the fact that the same B finding can be met in other ways (i.e., high *KIT* p.D816 V VAF ≥ 10% and/or MCs ≥ 30% in the BM biopsy). Also, the BST >20 ng/ml minor criterion seems to be less important in most cases of AdvSM and SSM as the major criterion is typically met. Lastly, the original use of BST ≥20 ng/ml, as opposed to other elevated BST values, appears to be based on limited data. Future discussion should consider what role BST values should play in SM diagnostic criteria. The value of using BST as a minor criterion may be highest in individuals with atypical *KIT* mutations when the major SM criterion is not met, when all other BM diagnostic testing is adequate, and the case has been reviewed by an experienced hematopathologist.

### BST monitoring in SM patients treated with tyrosine kinase inhibitors

Tryptase genotyping is important in the management of SM patients treated with TKIs. There have been several recent advances in the use of TKI treatments for SM. Three TKIs have been FDA approved for AdvSM including midostaurin, imatinib, and avapritinib. Tryptase genotypes of patients with AdvSM enrolled in these TKI trials were not assessed (83–87). Most patients with SM have a non-advanced subtype (BMM, ISM, or SSM). Low dose avapritinib at 25 mg daily was recently FDA approved for patients with ISM and is the first FDA approved treatment for patients with this SM subtype (88). Preliminary data from the PIONEER study shows a similar percentage of reduction in MC burden (i.e., serum tryptase and *KIT* p.D816V VAF) in patients with and without HαT treated with a low dose of the selective *KIT* p.D816V inhibitor avapritinib (89). There are several ongoing clinical trials including with avapritinib (NCT06327685, NCT03731260), bezuclastinib (NCT04996875, NCT05186753), elenestinib (NCT05609942, NCT04910685), and masitinib (NCT04333108).

Serial BST values, in addition to *KIT* p.D816V VAFs, measured in SM patients after a period of TKI treatment, may help guide when to repeat a BM biopsy to determine whether the SM neoplasm is in pathological remission. BST values are an indirect marker of BM MC burden. It is important to note that BST values in patients with SM likely represent a summation of the tryptase secreted by both neoplastic as well as wild-type MCs, and basophils to a lesser degree. BST values in SM patients, excluding those with SM-AHN where the AHN may also contribute to elevated BST values, who do not have HαT would be expected to fully normalize on a disease-modifying TKI therapy. In contrast, based on our experience, BST values in SM patients with HαT (excluding those with SM-AHN) assessed after a period of treatment with a disease-modifying TKI may not normalize BST values. Persistent BST elevations in patients with SM and unknown HαT status on TKI therapy are at significant risk. First, they risk effective therapy being discontinued under a false assumption that the TKI therapy is not effectively reducing the BST to “normal” values. Second, they risk unnecessary TKI dose escalations which are not indicated. Thus, tryptase genotyping is recommended for all patients with SM undergoing TKI treatment.

The half maximal inhibitory concentrations (IC_50_) of TKIs for wild type KIT and KIT D816V assessed in patients with SM have been previously reported and representative values are shown for a variety of TKIs in Table 4 (90–96). It is not clear what IC_50_ level and what dose of each specific TKI might lead to BST normalization in SM patients with HαT. Our experience has been that avapritinib 25 mg daily does not lead to normalization of BST values (BST <11.5 ng/ml) in patients with SM and HαT while it is too early to tell for other TKIs. Future studies should aim to determine whether each specific KIT inhibitor might lead to normalization of BST values in SM patients with and without HαT. Knowing whether to expect BST normalization or not would be helpful to guide when to perform an interval BM biopsy in these patients to assess for remission.

**Table 4:** Inhibitory activity of Various tyrosine kinase inhibitors.

TKI	IC_50_ WT KIT	IC_50_ KIT D816V	Reference(s)
Avapritinib	89.5 nM	3.1–13.0 nM	(92, 93)
Bezuclastinib	32.5 nM	3.4–14.0 nM	(92, 93)
Dasatinib	79.0 nM	37.0 nM	(94)
Elenestinib	82.6 nM	3.1–6.0 nM	(92, 93)
Imatinib	100 nM	> 10,000.0 nM	(95, 96)
Masitinib	200.0 nM	10,000.0 nM	(97)
Midostaurin	3.0–30.0 nM	100.0–300.0 nM	(98)
Nilotinib	30.0–300.0 nM	1,000.0–3,000.0 nM	(98)

IC_50_, half maximal inhibitory concentration; TKI, tyrosine kinase inhibitor; WT, wild type.

## Conclusions

Tryptase and tryptase genotyping assessments are essential in the screening, diagnosis, and management of SM. Patients with an elevated BST based upon their tryptase genotype should be offered a BM biopsy at a center with a hematopathologist expert to evaluate for SM. Tryptase genotyping as it relates to the WHO minor criterion BST >20 ng/ml, may be most important in the diagnosis of MMAS, BMM, and ISM, when there is minimal involvement of MCs in the BM such that the major criterion is not met. Tryptase genotyping is also needed to determine whether a patient with non-AdvSM has BMM if their BST is <125 ng/ml and whether the B-finding of BST >200 ng/ml is present. In patients with SM treated with a TKI, understanding a patient's BST value in relation to their tryptase genotype may help guide the decision on when to repeat a BM biopsy to assess for remission. Finally, ongoing clinical trials with selective TKIs should report on whether patients with SM and HαT have normalization or persistent elevation of BST values due to variable inhibition of wild type KIT and KIT D816V, as the potential BST nadir impacts the decision on when to pursue a BM biopsy.

## References

[1] Schwartz LB Lewis RA Austen KF. Tryptase from human pulmonary mast cells. Purification and characterization. J Biol Chem. (1981) 256:11939–43. 10.1016/S0021-9258(19)68496-6 7028744

[2] Schwartz LB Lewis RA Seldin D Austen KF. Acid hydrolases and tryptase from secretory granules of dispersed human lung mast cells. J Immunol. (1981) 126:1290–4. 10.4049/jimmunol.126.4.1290 7009736

[3] Glenner GG Cohen LA. Histochemical demonstration of a species-specific trypsin-like enzyme in mast cells. Nature. (1960) 185:846–7. 10.1038/185846a0 13828452

[4] Irani AA Schechter NM Craig SS DeBlois G Schwartz LB. Two types of human mast cells that have distinct neutral protease compositions. Proc Natl Acad Sci USA. (1986) 83:4464–8. 10.1073/pnas.83.12.4464 3520574 PMC323754

[5] He S-H Xie H. Modulation of tryptase secretion from human colon mast cells by histamine. World J Gastroenterol. (2004) 10:323–6. 10.3748/wjg.v10.i3.323 14760750 PMC4724929

[6] He S Gaça MD Walls AF. A role for tryptase in the activation of human mast cells: modulation of histamine release by tryptase and inhibitors of tryptase. J Pharmacol Exp Ther. (1998) 286:289–97. 10.1016/S0022-3565(24)37586-X 9655871

[7] Hallgren J Lindahl S Pejler G. Structural requirements and mechanism for heparin-dependent activation and tetramerization of human betaI- and betaII-tryptase. J Mol Biol. (2005) 345:129–39. 10.1016/j.jmb.2004.10.029 15567416

[8] Hallgren J Karlson U Poorafshar M Hellman L Pejler G. Mechanism for activation of mouse mast cell tryptase: dependence on heparin and acidic pH for formation of active tetramers of mouse mast cell protease 6. Biochemistry. (2000) 39:13068–77. 10.1021/bi000973b 11041873

[9] Hallgren J Bäckström S Estrada S Thuveson M Pejler G. Histidines are critical for heparin-dependent activation of mast cell tryptase. J Immunol. (2004) 173:1868–75. 10.4049/jimmunol.173.3.1868 15265919

[10] Sakai K Ren S Schwartz LB. A novel heparin-dependent processing pathway for human tryptase. Autocatalysis followed by activation with dipeptidyl peptidase I. J Clin Invest. (1996) 97:988–95. 10.1172/JCI118523 8613553 PMC507145

[11] Waters AM Park HJ Weskamp AL Mateja A Kachur ME Lyons JJ Elevated basal serum tryptase: disease distribution and variability in a regional health system. J Allergy Clin Immunol Pract. (2022) 10:2424–2435.e5. 10.1016/j.jaip.2021.12.031 35032694 PMC9273808

[12] Schwartz LB Sakai K Bradford TR Ren S Zweiman B Worobec AS The alpha form of human tryptase is the predominant type present in blood at baseline in normal subjects and is elevated in those with systemic mastocytosis. J Clin Invest. (1995) 96:2702–10. 10.1172/JCI118337 8675637 PMC185977

[13] Khoury JD Solary E Abla O Akkari Y Alaggio R Apperley JF The 5th edition of the world health organization classification of haematolymphoid tumours: myeloid and histiocytic/dendritic neoplasms. Leukemia. (2022) 36:1703–19. 10.1038/s41375-022-01613-1 35732831 PMC9252913

[14] Arber DA Orazi A Hasserjian RP Borowitz MJ Calvo KR Kvasnicka H-M International consensus classification of myeloid neoplasms and acute leukemias: integrating morphologic, clinical, and genomic data. Blood. (2022) 140:1200–28. 10.1182/blood.2022015850 35767897 PMC9479031

[15] Gotlib J Gerds AT Abdelmessieh P Ali H Castells M Dunbar A NCCN Guidelines® insights: systemic mastocytosis, version 3.2024. J Natl Compr Canc Netw. (2024) 22:8. 10.6004/jnccn.2024.0030 38862005

[16] Lyons JJ Yu X Hughes JD Le QT Jamil A Bai Y Elevated basal serum tryptase identifies a multisystem disorder associated with increased TPSAB1 copy number. Nat Genet. (2016) 48:1564–9. 10.1038/ng.3696 27749843 PMC5397297

[17] Chovanec J Tunc I Hughes J Halstead J Mateja A Liu Y Genetically defined individual reference ranges for tryptase limit unnecessary procedures and unmask myeloid neoplasms. Blood Adv. (2023) 7:1796–810. 10.1182/bloodadvances.2022007936 36170795 PMC10164828

[18] Trivedi NN Tong Q Raman K Bhagwandin VJ Caughey GH. Mast cell alpha and beta tryptases changed rapidly during primate speciation and evolved from gamma-like transmembrane peptidases in ancestral vertebrates. J Immunol. (2007) 179:6072–9. 10.4049/jimmunol.179.9.6072 17947681 PMC2366170

[19] Vanderslice P Ballinger SM Tam EK Goldstein SM Craik CS Caughey GH. Human mast cell tryptase: multiple cDNAs and genes reveal a multigene serine protease family. Proc Natl Acad Sci USA. (1990) 87:3811–5. 10.1073/pnas.87.10.3811 2187193 PMC53993

[20] Pallaoro M Fejzo MS Shayesteh L Blount JL Caughey GH. Characterization of genes encoding known and novel human mast cell tryptases on chromosome 16p13.3. J Biol Chem. (1999) 274:3355–62. 10.1074/jbc.274.6.3355 9920877

[21] Caughey GH Raymond WW Blount JL Hau LW Pallaoro M Wolters PJ Characterization of human gamma-tryptases, novel members of the chromosome 16p mast cell tryptase and prostasin gene families. J Immunol. (2000) 164:6566–75. 10.4049/jimmunol.164.12.6566 10843716

[22] Trivedi NN Raymond WW Caughey GH. Chimerism, point mutation, and truncation dramatically transformed mast cell delta-tryptases during primate evolution. J Allergy Clin Immunol. (2008) 121:1262–8. 10.1016/j.jaci.2008.01.019 18325577

[23] Wong GW Yasuda S Madhusudhan MS Li L Yang Y Krilis SA Human tryptase epsilon (PRSS22), a new member of the chromosome 16p13.3 family of human serine proteases expressed in airway epithelial cells. J Biol Chem. (2001) 276:49169–82. 10.1074/jbc.M108677200 11602603

[24] Wong GW Tang Y Feyfant E Sali A Li L Li Y Identification of a new member of the tryptase family of mouse and human mast cell proteases which possesses a novel COOH-terminal hydrophobic extension. J Biol Chem. (1999) 274:30784–93. 10.1074/jbc.274.43.30784 10521469

[25] Schwartz LB Min H-K Ren S Xia H-Z Hu J Zhao W Tryptase precursors are preferentially and spontaneously released, whereas mature tryptase is retained by HMC-1 cells, mono-Mac-6 cells, and human skin-derived mast cells. J Immunol. (2003) 170:5667–73. 10.4049/jimmunol.170.11.5667 12759448

[26] Trivedi NN Tamraz B Chu C Kwok P-Y Caughey GH. Human subjects are protected from mast cell tryptase deficiency despite frequent inheritance of loss-of-function mutations. J Allergy Clin Immunol. (2009) 124:1099–105.e1. 10.1016/j.jaci.2009.07.026 19748655 PMC2783561

[27] Marquardt U Zettl F Huber R Bode W Sommerhoff C. The crystal structure of human alpha1-tryptase reveals a blocked substrate-binding region. J Mol Biol. (2002) 321:491–502. 10.1016/s0022-2836(02)00625-3 12162961

[28] Selwood T Wang Z-M McCaslin DR Schechter NM. Diverse stability and catalytic properties of human tryptase alpha and beta isoforms are mediated by residue differences at the S1 pocket. Biochemistry. (2002) 41:3329–40. 10.1021/bi015662v 11876641

[29] Huang C Li L Krilis SA Chanasyk K Tang Y Li Z Human tryptases alpha and beta/II are functionally distinct due, in part, to a single amino acid difference in one of the surface loops that forms the substrate-binding cleft. J Biol Chem. (1999) 274:19670–6. 10.1074/jbc.274.28.19670 10391906

[30] Le QT Lyons JJ Naranjo AN Olivera A Lazarus RA Metcalfe DD Impact of naturally forming human α/β-tryptase heterotetramers in the pathogenesis of hereditary α-tryptasemia. J Exp Med. (2019) 216:2348–61. 10.1084/jem.20190701 31337736 PMC6780998

[31] Robey RC Wilcock A Bonin H Beaman G Myers B Grattan C Hereditary alpha-tryptasemia: UK prevalence and variability in disease expression. J Allergy Clin Immunol Pract. (2020) 8:3549–56. 10.1016/j.jaip.2020.05.057 32553831

[32] Glover SC Carter MC Korošec P Bonadonna P Schwartz LB Milner JD Clinical relevance of inherited genetic differences in human tryptases: hereditary alpha-tryptasemia and beyond. Ann Allergy Asthma Immunol. (2021) 127:638–47. 10.1016/j.anai.2021.08.009 34400315 PMC9413800

[33] Svetina M Šelb J Lyons JJ Korošec P Rijavec M. Clinically accessible amplitude-based multiplex ddPCR assay for tryptase genotyping. Sci Rep. (2024) 14:2416. 10.1038/s41598-024-52983-8 38287122 PMC10825142

[34] Alheraky A Wierenga ATJ Simpelaar A Hesp LB Minovic I Bagheri N Hereditary alpha tryptasemia: validation of a single-well Multiplex digital droplet PCR assay in a cohort of symptomatic patients. Clin Chem. (2024) 70:425–33. 10.1093/clinchem/hvad206 38073287

[35] Li O Hackney JA Choy DF Chang D Nersesian R Staton TL A targeted amplicon next-generation sequencing assay for tryptase genotyping to support personalized therapy in mast cell-related disorders. PLoS One. (2024) 19:e0291947. 10.1371/journal.pone.0291947 38335181 PMC10857577

[36] Wenzel S Irani AM Sanders JM Bradford TR Schwartz LB. Immunoassay of tryptase from human mast cells. J Immunol Methods. (1986) 86:139–42. 10.1016/0022-1759(86)90277-2 3511149

[37] Enander I Matsson P Nystrand J Andersson AS Eklund E Bradford TR A new radioimmunoassay for human mast cell tryptase using monoclonal antibodies. J Immunol Methods. (1991) 138:39–46. 10.1016/0022-1759(91)90062-k 2019745

[38] Schwartz LB Bradford TR Rouse C Irani AM Rasp G Van der Zwan JK Development of a new, more sensitive immunoassay for human tryptase: use in systemic anaphylaxis. J Clin Immunol. (1994) 14:190–204. 10.1007/BF01533368 7929694

[39] Hartmann K Escribano L Grattan C Brockow K Carter MC Alvarez-Twose I Cutaneous manifestations in patients with mastocytosis: consensus report of the European competence network on mastocytosis; the American academy of allergy, asthma & immunology; and the European academy of allergology and clinical immunology. J Allergy Clin Immunol. (2016) 137:35–45. 10.1016/j.jaci.2015.08.034 26476479

[40] Arock M Sotlar K Akin C Broesby-Olsen S Hoermann G Escribano L KIT Mutation analysis in mast cell neoplasms: recommendations of the European competence network on mastocytosis. Leukemia. (2015) 29:1223–32. 10.1038/leu.2015.24 25650093 PMC4522520

[41] Zanotti R Bonifacio M Isolan C Tanasi I Crosera L Olivieri F A multidisciplinary diagnostic approach reveals a higher prevalence of indolent systemic mastocytosis: 15-Years’ experience of the GISM network. Cancers (Basel). (2021) 13:1–13. 10.3390/cancers13246380 PMC8699786 34944999

[42] Cohen SS Skovbo S Vestergaard H Kristensen T Møller M Bindslev-Jensen C Epidemiology of systemic mastocytosis in Denmark. Br J Haematol. (2014) 166:521–8. 10.1111/bjh.12916 24761987

[43] Schuler CF Volertas S Khokhar D Yuce H Chen L Baser O Prevalence of mastocytosis and hymenoptera venom allergy in the United States. J Allergy Clin Immunol. (2021) 148:1316–23. 10.1016/j.jaci.2021.04.013 33895259

[44] Bergström A Hägglund H Berglund A Nilsson G Lambe M. Epidemiology of mastocytosis: a population-based study (Sweden). Acta Oncol. (2024) 63:44–50. 10.2340/1651-226X.2024.31406 38380845 PMC11332469

[45] Hermans MAW Rietveld MJA van Laar JAM Dalm VASH Verburg M Pasmans SGMA Systemic mastocytosis: a cohort study on clinical characteristics of 136 patients in a large tertiary centre. Eur J Intern Med. (2016) 30:25–30. 10.1016/j.ejim.2016.01.005 26809706

[46] Ungerstedt J Ljung C Klimkowska M Gülen T. Clinical outcomes of adults with systemic mastocytosis: a 15-year multidisciplinary experience. Cancers (Basel). (2022) 14:1–15. 10.3390/cancers14163942 PMC9405903 36010937

[47] Tiryaki TO Özkan SG Erdem S Aday AD Hindilerden İY Gelincik A Comprehensive mastocytosis data analysis from a single center. BMC Cancer. (2023) 23:82. 10.1186/s12885-022-10498-3 36694141 PMC9875486

[48] Sperr WR Kundi M Alvarez-Twose I van Anrooij B Oude Elberink JNG Gorska A International prognostic scoring system for mastocytosis (IPSM): a retrospective cohort study. Lancet Haematol. (2019) 6:e638–49. 10.1016/S2352-3026(19)30166-8 31676322 PMC7115823

[49] Sánchez-Muñoz L Alvarez-Twose I García-Montero AC Teodosio C Jara-Acevedo M Pedreira CE Evaluation of the WHO criteria for the classification of patients with mastocytosis. Mod Pathol. (2011) 24:1157–68. 10.1038/modpathol.2011.84 21552214

[50] Trizuljak J Sperr WR Nekvindová L Elberink HO Gleixner KV Gorska A Clinical features and survival of patients with indolent systemic mastocytosis defined by the updated WHO classification. Allergy. (2020) 75:1927–38. 10.1111/all.14248 32108361 PMC7115854

[51] Pieri L Bonadonna P Elena C Papayannidis C Grifoni FI Rondoni M Clinical presentation and management practice of systemic mastocytosis. A survey on 460 Italian patients. Am J Hematol. (2016) 91:692–9. 10.1002/ajh.24382 27060898

[52] Damaj G Joris M Chandesris O Hanssens K Soucie E Canioni D ASXL1 but not TET2 mutations adversely impact overall survival of patients suffering systemic mastocytosis with associated clonal hematologic non-mast-cell diseases. PLoS One. (2014) 9:e85362. 10.1371/journal.pone.0085362 24465546 PMC3897447

[53] Lim K-H Tefferi A Lasho TL Finke C Patnaik M Butterfield JH Systemic mastocytosis in 342 consecutive adults: survival studies and prognostic factors. Blood. (2009) 113:5727–36. 10.1182/blood-2009-02-205237 19363219

[54] Kennedy VE Perkins C Reiter A Jawhar M Lübke J Kluin-Nelemans HC Mast cell leukemia: clinical and molecular features and survival outcomes of patients in the ECNM registry. Blood Adv. (2022) 7:1713–24. 10.1182/bloodadvances.2022008292 PMC10182174 36094848

[55] Jennings SV Slee VM Zack RM Verstovsek S George TI Shi H Patient perceptions in mast cell disorders. Immunol Allergy Clin North Am. (2018) 38:505–25. 10.1016/j.iac.2018.04.006 30007467

[56] Jennings S Russell N Jennings B Slee V Sterling L Castells M The mastocytosis society survey on mast cell disorders: patient experiences and perceptions. J Allergy Clin Immunol Pract. (2014) 2:70–6. 10.1016/j.jaip.2013.09.004 24565772

[57] Russell N Jennings S Jennings B Slee V Sterling L Castells M The mastocytosis society survey on mast cell disorders: part 2-patient clinical experiences and beyond. J Allergy Clin Immunol Pract. (2019) 7:1157–1165.e6. 10.1016/j.jaip.2018.07.032 30098409

[58] van Anrooij B Kluin-Nelemans JC Safy M Flokstra-de Blok BMJ Oude Elberink JNG. Patient-reported disease-specific quality-of-life and symptom severity in systemic mastocytosis. Allergy. (2016) 71:1585–93. 10.1111/all.12920 27089859

[59] Mesa RA Sullivan EM Dubinski D Carroll B Slee VM Jennings SV Perceptions of patient disease burden and management approaches in systemic mastocytosis: results of the TouchStone healthcare provider survey. Cancer. (2022) 128:3700–8. 10.1002/cncr.34421 35996871 PMC9804550

[60] Brockow K Jofer C Behrendt H Ring J. Anaphylaxis in patients with mastocytosis: a study on history, clinical features and risk factors in 120 patients. Allergy. (2008) 63:226–32. 10.1111/j.1398-9995.2007.01569.x 18186813

[61] Gülen T Hägglund H Dahlén B Nilsson G. High prevalence of anaphylaxis in patients with systemic mastocytosis - a single-centre experience. Clin Exp Allergy. (2014) 44:121–9. 10.1111/cea.12225 24164252

[62] González de Olano D de la Hoz Caballer B Núñez López R Sánchez Muñoz L Cuevas Agustín M Diéguez MC Prevalence of allergy and anaphylactic symptoms in 210 adult and pediatric patients with mastocytosis in Spain: a study of the Spanish network on mastocytosis (REMA). Clin Exp Allergy. (2007) 37:1547–55. 10.1111/j.1365-2222.2007.02804.x 17883734

[63] van der Veer E van der Goot W de Monchy JGR Kluin-Nelemans HC van Doormaal JJ. High prevalence of fractures and osteoporosis in patients with indolent systemic mastocytosis. Allergy. (2012) 67:431–8. 10.1111/j.1398-9995.2011.02780.x 22229787

[64] Rossini M Zanotti R Bonadonna P Artuso A Caruso B Schena D Bone mineral density, bone turnover markers and fractures in patients with indolent systemic mastocytosis. Bone. (2011) 49:880–5. 10.1016/j.bone.2011.07.004 21782049

[65] Makovoz A Wang J Oshegbo G Park YH Lyons JJ Eisch AR Assessment of osteoporosis and fracture risk in mastocytosis within a north American cohort. J Allergy Clin Immunol Pract. (2021) 9:4459–4467.e10. 10.1016/j.jaip.2021.08.001 34403839

[66] Degboé Y Eischen M Nigon D Apoil P-A Mailhol C Tournier E Prevalence and risk factors for fragility fracture in systemic mastocytosis. Bone. (2017) 105:219–25. 10.1016/j.bone.2017.09.005 28919366

[67] McMurray JC Pacheco CS Schornack BJ Sun X Brunader JA Scott AE Standardized indolent systemic mastocytosis evaluations across a health care system: implications for screening accuracy. Blood. (2024) 144:408–19. 10.1182/blood.2023023347 38635793 PMC11418066

[68] Boggs NA Sun X Lyons JJ McMurray JC Rose DM Pryor EM Challenges in applying diagnostic criteria for systemic mastocytosis. Blood Adv. (2023) 7:3150–4. 10.1182/bloodadvances.2023009826 36848636 PMC10338204

[69] Šelb J Rijavec M Eržen R Zidarn M Kopač P Škerget M Routine KIT p.D816V screening identifies clonal mast cell disease in patients with hymenoptera allergy regularly missed using baseline tryptase levels alone. J Allergy Clin Immunol. (2021) 148:621–626.e7. 10.1016/j.jaci.2021.02.043 33753098 PMC10964493

[70] Jendoubi F Shourick J Negretto M Laurent C Apoil PA Evrard S Cutaneous mastocytosis in adults with a serum tryptase level < 20ng mL^-1^ : why we should investigate further. Br J Dermatol. (2021) 185:453–5. 10.1111/bjd.20098 33811770

[71] Alvarez-Twose I González de Olano D Sánchez-Muñoz L Matito A Esteban-López MI Vega A Clinical, biological, and molecular characteristics of clonal mast cell disorders presenting with systemic mast cell activation symptoms. J Allergy Clin Immunol. (2010) 125:1269–1278.e2. 10.1016/j.jaci.2010.02.019 20434205

[72] Alvarez-Twose I González-de-Olano D Sánchez-Muñoz L Matito A Jara-Acevedo M Teodosio C Validation of the REMA score for predicting mast cell clonality and systemic mastocytosis in patients with systemic mast cell activation symptoms. Int Arch Allergy Immunol. (2012) 157:275–80. 10.1159/000329856 22042301

[73] Berezowska S Flaig MJ Ruëff F Walz C Haferlach T Krokowski M Adult-onset mastocytosis in the skin is highly suggestive of systemic mastocytosis. Mod Pathol. (2014) 27:19–29. 10.1038/modpathol.2013.117 23807778

[74] Tanasi I Crosera L Taus F Orsolini G Adami G Olivieri F Underlying systemic mastocytosis in patients with unexplained osteoporosis: score proposal. Bone. (2024) 186:117141. 10.1016/j.bone.2024.117141 38823568 PMC11854854

[75] Keyzer JJ de Monchy JG van Doormaal JJ van Voorst Vader PC. Improved diagnosis of mastocytosis by measurement of urinary histamine metabolites. N Engl J Med. (1983) 309:1603–5. 10.1056/NEJM198312293092603 6646186

[76] Morrow JD Guzzo C Lazarus G Oates JA Roberts LJ. Improved diagnosis of mastocytosis by measurement of the major urinary metabolite of prostaglandin D2. J Invest Dermatol. (1995) 104:937–40. 10.1111/1523-1747.ep12606209 7769262

[77] Divekar R Butterfield J. Urinary 11β-PGF2α and N-methyl histamine correlate with bone marrow biopsy findings in mast cell disorders. Allergy. (2015) 70:1230–8. 10.1111/all.12668 26095439

[78] Lueke AJ Meeusen JW Donato LJ Gray AV Butterfield JH Saenger AK. Analytical and clinical validation of an LC-MS/MS method for urine leukotriene E4: a marker of systemic mastocytosis. Clin Biochem. (2016) 49:979–82. 10.1016/j.clinbiochem.2016.02.007 26908217

[79] Valent P Horny HP Escribano L Longley BJ Li CY Schwartz LB Diagnostic criteria and classification of mastocytosis: a consensus proposal. Leuk Res. (2001) 25:603–25. 10.1016/s0145-2126(01)00038-8 11377686

[80] Valent P Hartmann K Hoermann G Reiter A Alvarez-Twose I Brockow K Harmonization of diagnostic criteria in mastocytosis for use in clinical practice: WHO vs ICC vs AIM/ECNM. J Allergy Clin Immunol Pract. (2024) 12:3250–60. 10.1016/j.jaip.2024.08.044 39216803

[81] Sotlar K Horny H-P Simonitsch I Krokowski M Aichberger KJ Mayerhofer M CD25 Indicates the neoplastic phenotype of mast cells: a novel immunohistochemical marker for the diagnosis of systemic mastocytosis (SM) in routinely processed bone marrow biopsy specimens. Am J Surg Pathol. (2004) 28:1319–25. 10.1097/01.pas.0000138181.89743.7b 15371947

[82] Shean RC Hellwig S Saadalla A George TI Rets AV. High-sensitivity KIT D816V variation analysis by droplet digital polymerase chain reaction: the reference laboratory perspective. Am J Clin Pathol. (2025):1–5. 10.1093/ajcp/aqaf008 40036308

[83] Gotlib J Kluin-Nelemans HC George TI Akin C Sotlar K Hermine O Efficacy and safety of midostaurin in advanced systemic mastocytosis. N Engl J Med. (2016) 374:2530–41. 10.1056/NEJMoa1513098 27355533

[84] DeAngelo DJ George TI Linder A Langford C Perkins C Ma J Efficacy and safety of midostaurin in patients with advanced systemic mastocytosis: 10-year median follow-up of a phase II trial. Leukemia. (2018) 32:470–8. 10.1038/leu.2017.234 28744009

[85] Gotlib J Reiter A Radia DH Deininger MW George TI Panse J Efficacy and safety of avapritinib in advanced systemic mastocytosis: interim analysis of the phase 2 PATHFINDER trial. Nat Med. (2021) 27:2192–9. 10.1038/s41591-021-01539-8 34873345 PMC8674139

[86] DeAngelo DJ Radia DH George TI Robinson WA Quiery AT Drummond MW Safety and efficacy of avapritinib in advanced systemic mastocytosis: the phase 1 EXPLORER trial. Nat Med. (2021) 27:2183–91. 10.1038/s41591-021-01538-9 34873347 PMC8674134

[87] Alvarez-Twose I Matito A Morgado JM Sánchez-Muñoz L Jara-Acevedo M García-Montero A Imatinib in systemic mastocytosis: a phase IV clinical trial in patients lacking exon 17 KIT mutations and review of the literature. Oncotarget. (2017) 8:68950–63. 10.18632/oncotarget.10711 28978170 PMC5620310

[88] Gotlib J Castells M Elberink HO Siebenhaar F Hartmann K Broesby-Olsen S Avapritinib versus placebo in indolent systemic mastocytosis. NEJM evid. (2023) 2:1–15. 10.1056/EVIDoa2200339 38320129

[89] Castells M Akin C Maurer M Hartmann K Broesby-Olsen S George TI Analysis of hereditary alpha tryptasemia and AssociationWith baseline characteristics in patients with IndolentSystemic mastocytosis enrolled on the PIONEER study. American Initiative in Mast Cell Diseases (AIM) Conference; Boston, MA; May 18–19, 2024. (2024). p. 1–10.

[90] Guarnieri AL. Preclinical data identifies bezuclastinib as a differentiated KIT inhibitor with unique selectivity to KIT D816V and minimal evidence of brain penetration. Mol Cancer Ther. (2021) 20(12_Supplement):1–10. 10.1158/1535-7163.TARG-21-P257

[91] Tashi T Hermine O Castells M Guilarte M Sabato V Maurer M Elenestinib, an investigational, next generation KIT D816V inhibitor, reduces mast cell burden, improves symptoms, and has a favorable safety profile in patients with indolent systemic mastocytosis: analysis of the harbor trial. Blood. (2023) 142:76–76. 10.1182/blood-2023-188904

[92] Shah NP Lee FY Luo R Jiang Y Donker M Akin C. Dasatinib (BMS-354825) inhibits KITD816V, an imatinib-resistant activating mutation that triggers neoplastic growth in most patients with systemic mastocytosis. Blood. (2006) 108:286–91. 10.1182/blood-2005-10-3969 16434489

[93] Heinrich MC Griffith DJ Druker BJ Wait CL Ott KA Zigler AJ. Inhibition of c-kit receptor tyrosine kinase activity by STI 571, a selective tyrosine kinase inhibitor. Blood. (2000) 96:925–32. 10.1182/blood.V96.3.925 10910906

[94] Growney JD Clark JJ Adelsperger J Stone R Fabbro D Griffin JD Activation mutations of human c-KIT resistant to imatinib mesylate are sensitive to the tyrosine kinase inhibitor PKC412. Blood. (2005) 106:721–4. 10.1182/blood-2004-12-4617 15790786 PMC1895184

[95] Dubreuil P Letard S Ciufolini M Gros L Humbert M Castéran N Masitinib (AB1010), a potent and selective tyrosine kinase inhibitor targeting KIT. PLoS One. (2009) 4:e7258. 10.1371/journal.pone.0007258 19789626 PMC2746281

[96] Gleixner KV Mayerhofer M Aichberger KJ Derdak S Sonneck K Böhm A PKC412 inhibits *in vitro* growth of neoplastic human mast cells expressing the D816V-mutated variant of KIT: comparison with AMN107, imatinib, and cladribine (2CdA) and evaluation of cooperative drug effects. Blood. (2006) 107:752–9. 10.1182/blood-2005-07-3022 16189265

[97] Lyons JJ Yi T. Mast cell tryptases in allergic inflammation and immediate hypersensitivity. Curr Opin Immunol. (2021) 72:94–106. 10.1016/j.coi.2021.04.001 33932709

[98] Fukuoka Y Schwartz LB. Active monomers of human beta-tryptase have expanded substrate specificities. Int Immunopharmacol. (2007) 7:1900–8. 10.1016/j.intimp.2007.07.007 18039527 PMC2278033

